# Dunning-Kruger Effect: Intuitive Errors Predict Overconfidence on the Cognitive Reflection Test

**DOI:** 10.3389/fpsyg.2021.603225

**Published:** 2021-04-08

**Authors:** Mariana V. C. Coutinho, Justin Thomas, Alia S. M. Alsuwaidi, Justin J. Couchman

**Affiliations:** ^1^Department of Psychology, College of Natural Health and Sciences, Zayed University, Abu Dhabi, United Arab Emirates; ^2^Department of Psychology, Albright College, Reading, PA, United States

**Keywords:** Dunning-Kruger Effect, overconfidence, cognitive reflection test, answer fluency, reasoning, Type 1 and Type 2 processes

## Abstract

The Cognitive Reflection Test (CRT) is a measure of analytical reasoning that cues an intuitive but incorrect response that must be rejected for successful performance to be attained. The CRT yields two types of errors: Intuitive errors, which are attributed to Type 1 processes; and non-intuitive errors, which result from poor numeracy skills or deficient reasoning. Past research shows that participants who commit the highest numbers of errors on the CRT overestimate their performance the most, whereas those with the lowest error-rates tend to slightly underestimate. This is an example of the Dunning-Kruger Effect (DKE). The present study examined how intuitive vs. non-intuitive errors contribute to overestimation in the CRT at different levels of performance. Female undergraduate students completed a seven-item CRT test and subsequently estimated their raw score. They also filled out the Faith in Intuition (FI) questionnaire, which is a dispositional measure of intuitive thinking. Data was separated into quartiles based on level of performance on the CRT. The results demonstrated the DKE. Additionally, intuitive and non-intuitive errors predicted miscalibration among low, but not high performers. However, intuitive errors were a stronger predictor of miscalibration. Finally, FI was positively correlated with CRT self-estimates and miscalibration, indicating that participants who perceived themselves to be more intuitive were worse at estimating their score. These results taken together suggest that participants who perform poorly in the CRT and also those who score higher in intuitive thinking disposition are more susceptible to the influences of heuristic-based cues, such as answer fluency, when judging their performance.

## Introduction

People overestimate their level of skill, knowledge, and performance in a variety of contexts (Dunning et al., 2003; De Bruin et al., 2017; Sanchez and Dunning, 2018). Miscalibration between estimated and actual performance follows a consistent pattern. People with the lowest scores on a test tend to show the highest overestimations of their performance, midrange performers show less overestimation, and the best performers tend to slightly underestimate themselves (e.g., Kruger and Dunning, 1999; Burson et al., 2006; Ehrlinger et al., 2008; Moore and Healy, 2008; Pennycook et al., 2017). This phenomenon is known as the Dunning-Kruger Effect (DKE). It has been the focus of much research in a large part due to what it implies about self-regulated learning. Not knowing the extent to which one lacks knowledge or skills within a particular domain can lead to the implementation of suboptimal strategies during study, which in turn may hinder learning and performance. And worse, the DKE may prevent people from overcoming deficits in knowledge or skills because one of the prerequisites for self-improvement is the recognition of one's own shortcomings. Although the link between performance level and calibration accuracy is well-established in the literature, the reasons why low performers show greater levels of overconfidence is yet not well-understood. To shed light on this, the present study explores the DKE within the context of a reflective-reasoning test, where a compelling, intuitive but incorrect response must be overridden for successful performance to be attained.

Initial exploration of the DKE focused on tests of grammar, logical reasoning, and humor (Kruger and Dunning, 1999). It was hypothesized that people with deficits in knowledge or skills in a particular domain would be less likely to recognize those deficits, and therefore would be more likely to overestimate their performance. To test the hypothesis, in three separate studies, participants completed a task that measured their knowledge of American Standard Written English, their ability to analyze and evaluate arguments, and their ability to identify good jokes. Upon completion of each task participants were prompted to estimate their raw score as well as how well they did relative to their peers. Kruger and Dunning separated their data into quartiles based on participants' actual performance, and compared that performance to their assessments of themselves. This analysis yielded a common pattern across all three studies. Participants in the bottom quartile overestimated their performance in terms of absolute and relative judgments, whereas those in the upper quartile underestimated. However, it was not only the direction of miscalibration that differed between the bottom and upper quartiles. Miscalibration was much greater for low performers, indicating that low performers are the ones least aware of how much they actually know.

Many other studies have reported similar findings in various domains, including knowledge-based tests like geography (Ehrlinger and Dunning, 2003), skill-based tests of driving (Marottoli and Richardson, 1998), card gaming (Simons, 2013), sport coaching (Sullivan et al., 2019), and ability-based tests of emotional intelligence (Sheldon et al., 2014). Kruger and Dunning (1999) posited that low performers overestimate because accurate self-assessment depends on the same set of skills and knowledge as performance. In their view, deficits in on-line metacognitive monitoring, due to limited knowledge or skills, are the main cause of overestimation. Such deficits hinder participants' ability to discriminate incorrect from correct responses, causing errors to go undetected. Dunning's (2011) summarizes this by stating that because low performers are “operating from incomplete and corrupted knowledge, they would make many mistakes and not recognize those mistakes as they made them” (p. 260). Such inability to identify ones' own errors in turn causes low performers to believe they performed much better than they actually did, leading to poorly-calibrated self-estimates. This theoretical account of the DKE is known as the double-burden hypothesis because limited knowledge places two burdens on individuals: it prevents them from producing correct responses and also from recognizing when their responses were incorrect. Additional explanations of the DKE have also been proposed. For example, researchers have suggested that regression to mean could elicit similar patterns of overestimation (Krueger and Mueller, 2002; Feld et al., 2017). But studies showed that this artifact can only explain part of the effect, not all (see Ehrlinger et al., 2008). In support of Kruger and Dunning's (1999) account of the DKE, evidence suggests that metacognitive differences in on-line monitoring exist between low and high performers (McIntosh et al., 2019). Yet, the reasons why limited knowledge is associated with one's inability to recognize one's own mistakes, and whether failings in on-line metacognitive monitoring are the main driver of overestimation are still not clear, and therefore deserve further investigation.

Recently, researchers started utilizing a performance-based measure of analytical reasoning, the Cognitive Reflection Test (CRT) to explore the DKE within the context of dual-process theories of cognition (Pennycook et al., 2017). Dual-process theories hold that humans have two distinct modes of thinking: Type 1 and Type 2 processing modes (Evans and Stanovich, 2013). Type 1 processes are characterized as fast, effortless, and heuristic-based, and its outputs are intuitions. In contrast, Type 2 processes are slower, effortful, rule-based and have the potential to override Type 1 outputs. Although humans are capable of engaging in both types of processes, research shows that individuals differ in their willingness and propensity to engage in Type 2 processing (Pennycook et al., 2015a). Some people are more likely to rely on Type 2 processes to solve problems than others. Such individuals are classified as analytical thinkers, whereas those who are more likely to engage in Type 1 processing are referred as intuitive thinkers. A range of benefits has been associated with the analytical cognitive style, including heightened metacognitive monitoring during reasoning (Mata et al., 2013; Thompson and Johnson, 2014; Pennycook et al., 2015b). For example, Mata and colleagues showed that analytic thinkers are more accurate in their estimates of performance than intuitive individuals. These findings are in line with Kruger and Dunning's (1999) account of the DKE. They also point toward the importance of examining the role of Type I mode of processing in overconfidence to better understand the DKE. The current study attempted to do that using the CRT.

The original CRT is a three-item measure of analytical reasoning developed by Frederick (2005). Each question is structured in such way that cues an easily accessible, compelling, but incorrect intuitive answer. Successful performance in the CRT thus depends on participants' capacity and willingness to engage in analytical processing to overcome the initial intuitive response. For example, consider the following widely known CRT problem: “A bat and a ball cost $1.10 in total. The bat costs $1.00 more than the ball. How much does the ball cost? ___cents.” The answer that readily comes to mind is 10, which is both intuitive and incorrect. The correct answer is 5 cents ($.05 ball + $1.05 bat = $1.10), which is attained through deliberative reasoning intervention. In this case, X + (X + 1) = 1.10, solve for X. As participants reflect upon the intuitive answer, a conflict arises between the intuitive and the analytical solution, prompting them to reject the intuitive answer and accept the analytical one.

It is important to add that participants' responses to the CRT fall into three categories: analytical-correct, intuitive-incorrect, and non-intuitive-incorrect solutions. In the example above, “5 cents” is the analytical-correct solution whereas “10 cents” is an intuitive-incorrect solution. Non-intuitive-incorrect on the other hand would be any response that is neither correct nor match the intuitive-incorrect solution. The correct solution is elicited through the intervention of analytical or Type 2 processing. Conversely, intuitive responses are the product of Type 1 processing, and lastly non-intuitive-incorrect responses result from poor numeracy skills or deficient reasoning (Hertzog et al., 2018). In addition to Frederick's (2005) three-item CRT, there are newer versions of the CRT, including Toplak's et al. (2014) seven-item CRT and Thomson and Oppenheimer's (2016) four-item CRT.

Because the CRT cues a reliable but incorrect intuitive response, it has the potential to shed light into how mistakes elicited by Type 1 processes contribute to overconfidence particularly in relation to other, more general mistakes. To date, however, only one study examined the DKE utilizing the CRT. Pennycook et al. (2017) created an 8-item CRT by combining Toplak's et al. (2014) four-item CRT with Thomson and Oppenheimer's (2016) four-item CRT. Participants in their study first completed the 8-item CRT and then estimated their raw score in the test. The results showed that less-analytic individuals—that is, participants with the lowest scores (0–2 points) greatly overestimated their performance. Their mean estimated score was 4.78 whereas the average number of correct responses was 1.42. Their degree of miscalibration was therefore 3.4 points, which was much higher than 1.1 observed in high performers. In summary, Pennycook et al. (2017) replicated the DKE using the CRT and by doing so demonstrated that less analytic-individuals are the ones who appear to be the least aware of their reasoning abilities, as indicated by the large discrepancy between actual and estimated scores.

Pennycook and colleagues did not examine the role of Type 1 outputs, intuitive responses, on overestimation. Understanding the influences of intuitive responses on miscalibration is important because it can help unveil the mechanisms mediating the DKE, particularly why low performers have difficulty recognizing their own mistakes, and therefore greatly overestimate their performance (Kruger and Dunning, 1999; Dunning, 2011). Intuitive responses differ from the other two responses in terms of answer fluency—that is, the speed or ease by which an answer is generated (Pennycook et al., 2015a). They are produced more fluently (quickly) than analytical-correct and non-intuitive-incorrect responses. Additionally, previous studies have shown that variations in the speed in which an answer is generated predicts how confident participants are on the correctness of their answer (Thompson et al., 2011, 2013). For example, in Thompson et al. (2011), participants were presented with a reasoning task, instructed to give the first answer came to mind to each question, and right after generating a response rate their feeling of rightness on the answer provided—that is rate how certain they were their responses were in fact correct. The results showed a robust negative correlation between the amount of time taken to respond and feeling of rightness. The shorter was the time to respond, the more confident they were in the correctness of their responses, indicating that participants utilize answer fluency as cue for constructing local, metacognitive judgments. Given that answer fluency is predictive of feeling of rightness, and general estimates of performance are judgments based on how many items participants believe they answered correctly, it is reasonable to assume that answer fluency may also influence general estimates of performance. Such influence may occur because answers that are generated more fluently, and thus accompanied by stronger feeling of rightness, are more likely to be counted as correct when participants estimate their total score in a test.

Thompson et al. (2011) showed that increases in answer fluency influence confidence in the accuracy of a response. The higher the fluency, the more confident participants were that a response was correct. This effect was observed even though there were no differences in objective accuracy across responses. All intuitive responses were incorrect. This shows that participants utilized fluency as a cue for judging the accuracy of a response even though such cue was not predictive of performance. Considering these findings and Ackerman and Leiser's (2014) report that low performing students are more likely to rely on unreliable cues when assessing the correctness of their answers, we propose that low performers' difficulty in recognizing their own mistakes may result from overreliance on fluency as a cue for local, metacognitive judgments. That is, low performers may be more likely than higher performers to interpret increases in fluency as an indication that a response is correct. Because of that, low performers end up not recognizing their own errors, and therefore overestimating their overall performance. If indeed having limited knowledge and skills increases one's susceptibility to the influences of fluency, we would expect that low performers would be less likely to recognize they made a mistake when the response was intuitive than when it was non-intuitive-incorrect. Hence, intuitive responses should be a stronger predictor of overestimation than non-intuitive incorrect responses. On the other hand, such a pattern is not expected among high performers, or at least not to the same degree. This prediction is in line with Mata's, Ferreira and Sherman (2013) suggestion that more-analytic thinkers, like those who perform well on the CRT, naturally initiate Type 2 processing to verify the accuracy of intuitive responses.

In the present study, our aim was to investigate the relationship between intuitive responses and calibration accuracy at different performance levels on the CRT. Participants completed the CRT and then estimated their performance. They also filled out the Faith in Intuition scale, which is a self-report measure of intuitive thinking disposition. We wanted to know if self-reported intuitive thinking disposition influences estimates of performance. Participants were separated into quartiles based on their objective performance on the CRT, and the average number of intuitive-incorrect, non-intuitive-incorrect and correct responses were calculated per participant per quartile. Actual CRT scores were subtracted from estimated CRT scores to calculate calibration accuracy. We tested the hypothesis that intuitive responding contributes to miscalibration among low performers but not high performers. We also hypothesized that intuitive-incorrect responses are a stronger predictor of overestimation than non-intuitive-incorrect responses among low performers, and that increased faith in intuition leads to increased miscalibration.

## Methods

### Participants

Participants were recruited through an email sent by faculty members to undergraduate students registered in psychology courses at Zayed University, Abu Dhabi campus. One-hundred and seventy-eight undergraduate female students enrolled in psychology courses at Zayed Universityparticipated in the study. Participants with missing data were excluded from the data analysis. There were nine participants who did not provide an estimate of their CRT performance, and therefore were excluded. A total of 169 participants were included in the data analysis. Participants ages ranged from 18 to 32 with a mean of 21. Most participants were from the United Arab Emirates (155), and the remaining were from Yemen (*N* = 4), Oman (*N* = 4), Bahrain (*N* = 3), Eritrea (*N* = 2), and Somalia (*N* = 1). Most participants were majoring in psychology (*N* = 160) while a few came from other majors, including business (*N* = 1), communication (*N* = 4), marketing (*N* = 3), and nutrition (*N* = 1). Participants were all bilingual in English and Arabic. The institutional language is English. The study received ethical approval from the institution's research ethics committee (R17101). All participants gave written informed consent prior to the study's commencement.

### Materials

#### Cognitive Reflection Test

The CRT measures analytical thinking. Each problem in the CRT cues an intuitive-incorrect response, which participants are supposed to override to reach the correct solution. There are three well-known versions of the CRT. The original CRT consists of three questions developed by Frederick (2005). An extended version of it was then developed by Toplak et al. (2014) that included four more four new items. Lastly, because the two CRTs had been widely used, Thomson and Oppenheimer (2016) developed a new one including four questions. We opted not to use Frederick's 3-item CRT because of its wide use in research and in class demonstrations. Instead, similar to Pennycook et al. (2017), we combined two versions of the CRT, Toplak's et al. (2014) 4-new items and Thomson and Oppenheimer's (2016) CRT-2. However, the fourth item of CRT-2 was not included because of participants' lack of familiarity with some of the terms covered in the question. In total, the test had seven items as shown below:

- If John can drink one barrel of water in 6 days, and Mary can drink one barrel of water in 12 days, how long would it take them to drink one barrel of water together? _____ days (correct answer: 4 days; intuitive answer: 9).
- Jerry received both the 15th highest and the 15th lowest mark in the class. How many students are in the class? ______ students (correct answer: 29 students; intuitive answer: 30).
- A man buys a pig for $60, sells it for $70, buys it back for $80, and sells it finally for $90. How much has he made? _____ dollars (correct answer: $20; intuitive answer: $10].
- Simon decided to invest $8,000 in the stock market 1 day early in 2008. Six months after he invested, on July 17, the stocks he had purchased were down 50%. Fortunately for Simon, from July 17 to October 17, the stocks he had purchased went up 75%. At this point, Simon has: a. broken even in the stock market, b. is ahead of where he began, c. has lost money (correct answer: c, because the value at this point is $7,000; intuitive response: b).
- If you're running a race and you pass the person in second place, what place are you in? (correct answer: second; intuitive answer: first).
- A farmer had 15 sheep and all but eight died. How many are left? (correct answer: 8; intuitive answer: 7).
- Emily's father has three daughters. The first two are named April and May. What is the third daughter's name? (correct answer: Emily; intuitive answer: June).

The estimate of internal consistency (Cronbach's alpha) for all seven items was 0.36. The low level of internal consistency could be the result of lack of homogeneity among items from the two scales or the small number of items. Each of the questions bring about three possible solutions. Participants responses were coded as correct if they matched the correct solution, and as intuitive-incorrect if they corresponded to the intuitive solution. Participants could also give a response that was neither intuitive-incorrect nor correct. Such answers are coded as non-intuitive-incorrect.

#### Faith in Intuition

Faith in Intuition (FI) is a self-report measure of intuitive thinking disposition developed by Pacini and Epstein (1999). It consists of two subscales: Experiential Ability (E-A) and Experiential Engagement (E-E), each containing 10 items (see survey in Supplementary Material). Experiential Ability measures confidence level on intuitive impressions and feelings. Experiential Engagement measures reliance on and enjoyment on intuition in decision making. Each statement is rated by participants from 1 to 5 according to how well it applies to them; where 1 indicates completely false and 5 indicates completely true. The full FI's internal reliability in the current study was acceptable, α = 0.77.

### Design and Procedure

Upon arrival in the laboratory, participants were greeted by a research assistant and directed to a workstation with a computer. After giving informed consent to participate in the study, participants filled out a demographic questionnaire and subsequently received detailed instructions about how to complete the CRT. The CRT included seven questions, and they were presented to participants on paper. After completing the last question in the test, participants were asked to evaluate their performance by answering the following question: “how many of the seven items do you think you answered correctly?” Lastly, they filled out the FI questionnaire.

### Data Analysis

To investigate degree of overestimation (or underestimation), we calculated a calibration score (estimated score—actual score) for each student. Positive values correspond to overestimation and negative to underestimation. The absolute value of the difference score, or calibration accuracy, was also calculated, in order to examine the accuracy of the performance judgments. Calibration accuracy was computed as |estimated performance–actual performance|. The ideal score would be 0.

To evaluate the main hypotheses of the present study, a series of regression analysis were conducted using IBM SPSS Statistics, version 26. Specifically, multiple regressions were carried out to evaluate whether intuitive and non-intuitive errors predict miscalibration across participants at different levels of performance, as well as for the whole sample. Predictor variables were checked for multicollinearity. Single regressions were also performed to evaluate the predictive value of combined intuitive and correct CRT scores in predicting estimated CRT. Finally, additional single regressions were conducted to probe FI contributions to estimated CRT scores and miscalibration.

## Results

### Overestimation

Participants overestimated their score on the CRT. The mean estimated score was 4.85 out of 7 (*SD* = 1.09), whereas the average number of correct responses was 1.78 (*SD* = 1.23), *t*_(168)_ = 24.8, *p* < 0.001. Furthermore, participants made significantly more intuitive-incorrect responses (*M* = 3.21, *SD* = 1.29) than correct responses (*M* = 1.78, *SD* = 1.23), *t*_(168)_ = 7.98, *p* < 0.001. Descriptive statistics for correct, intuitive, and non-intuitive incorrect responses per item in the CRT can be found on Supplementary Table 1.

### Dunning-Kruger Effect

To explore the accuracy of estimated scores across different levels of objective performance, a quartile-split based on actual performance in the CRT was used. Mean of actual and estimated scores for each quartile and its sample size are shown on Table 1. As a manipulation check, a quartile Analysis of Variance (ANOVA) on actual performance was performed. Actual scores differed significantly across quartile, *p* < 0.001.

**Table 1:** Descriptive Statistics [*M* (*SD*)] for actual CRT scores, estimated CRT per quartiles.

**Quartile**	***N***	**CRT scores** ***M (SD)***	**CRT estimates** ***M (SD)***
1	70	0.56 (0.5)	4.81 (1.13)
2	50	2.00 (0)	4.94 (0.98)
3	37	3.00 (0)	4.68 (1.18)
4	12	4.25 (0.62)	5.25 (1.05)

**Figure 1:**
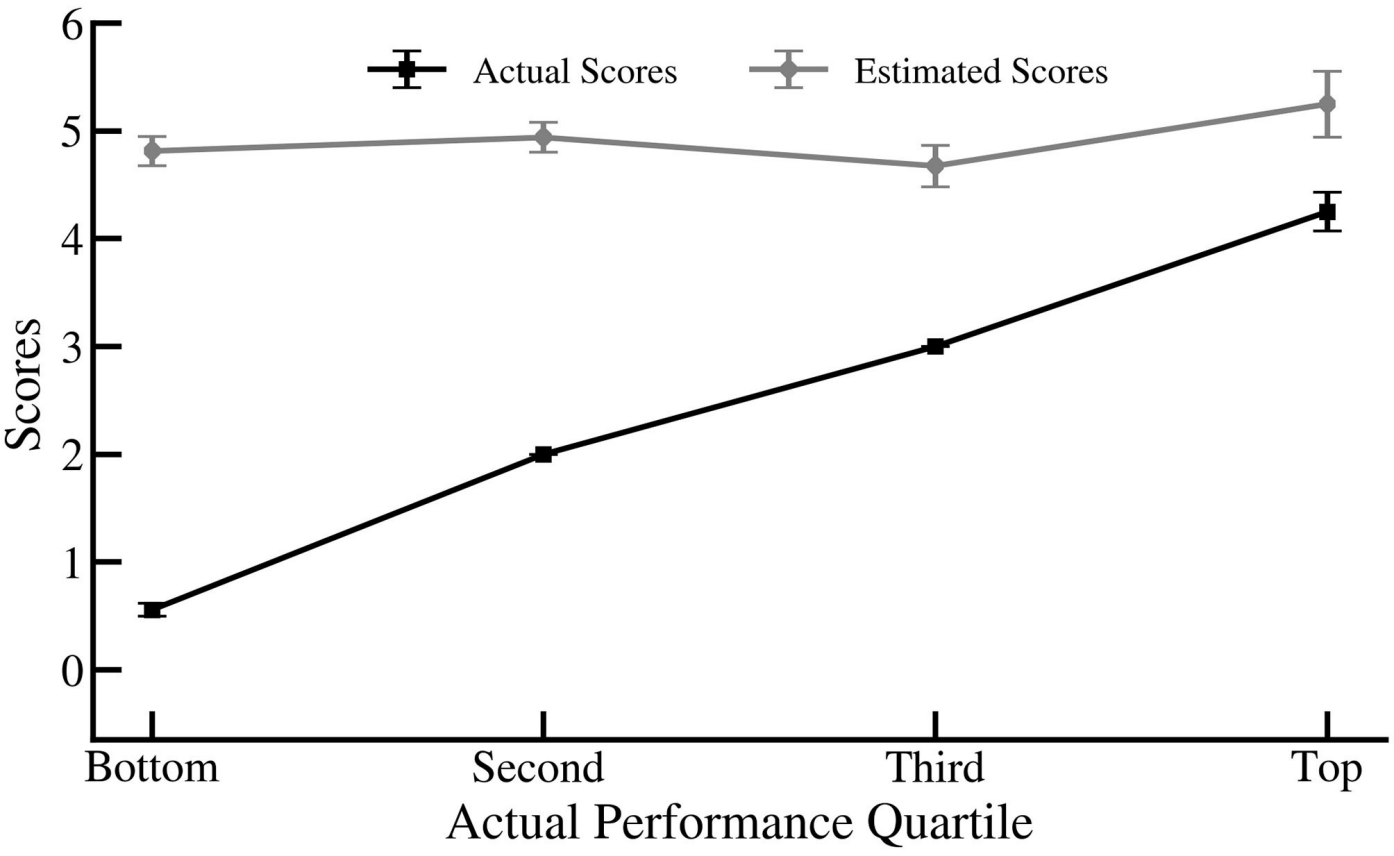
Estimated and actual scores for each performance quartile. Error bars indicate standard error.

To evaluate whether the difference between estimated and actual scores varied across students at different quartiles, we conducted a mixed-design ANOVA. The analysis yielded an interaction between quartile and the difference between estimated and actual scores, [*F*_(3,165)_ = 57.73], *p* < 0.001, η^2^ = 0.512. A Tukey Honest Significant Difference (HSD) *post-hoc* test indicated that the difference between estimated and actual scores was significantly higher for bottom performers than for second (*p* < 0.001), third (*p* < 0.001) and upper quartile (*p* < 0.001) groups. As Figure 1 shows, this difference decreased with an increase in performance quartile. Students in the bottom quartile (*M* = *0.56, SD* = *0.5)* on average estimated that they had a score of *4.81 (SD* = *1.13)*, overestimating their performance by 4.26 points, *t*_(69)_ = 29.68, *p* < 0.001. Similarly, students in the lower and upper middle quartile overestimated their performance by 2.9, *t*_(49)_ = 21.67, *p* < 0.001 and 1.7, *t*_(36)_ = 8.63, *p* < 0.001. Whereas, high performers overestimated (*M* = 5.25, *SD* = 1.05) their performance (*M* = 4.25, *SD* = 0.62), by only 1 point, *t*_(11)_ = 2.87, *p* = 0.015.

To be able to compare the magnitude of miscalibration between participants in the top and bottom quartiles, we followed the Burson et al. (2006) method and coded miscalibration as [estimated performance—actual performance] for lowest performers and [actual performance—estimated performance] for the top quartile. This transformation is important when comparing miscalibration between groups because it gives the means the same sign while maintaining the variance around the means. Notably, the magnitude of the estimation error was greater for low (*M* = 4.26, *SD* = 1.2) than high performers (*M* = 1.3, *SD* = 0.78), *t*_(80)_ = 8.13, *p* < 0.001, replicating the DKE.

### Error Type Comparison Across Quartiles

**Figure 2:**
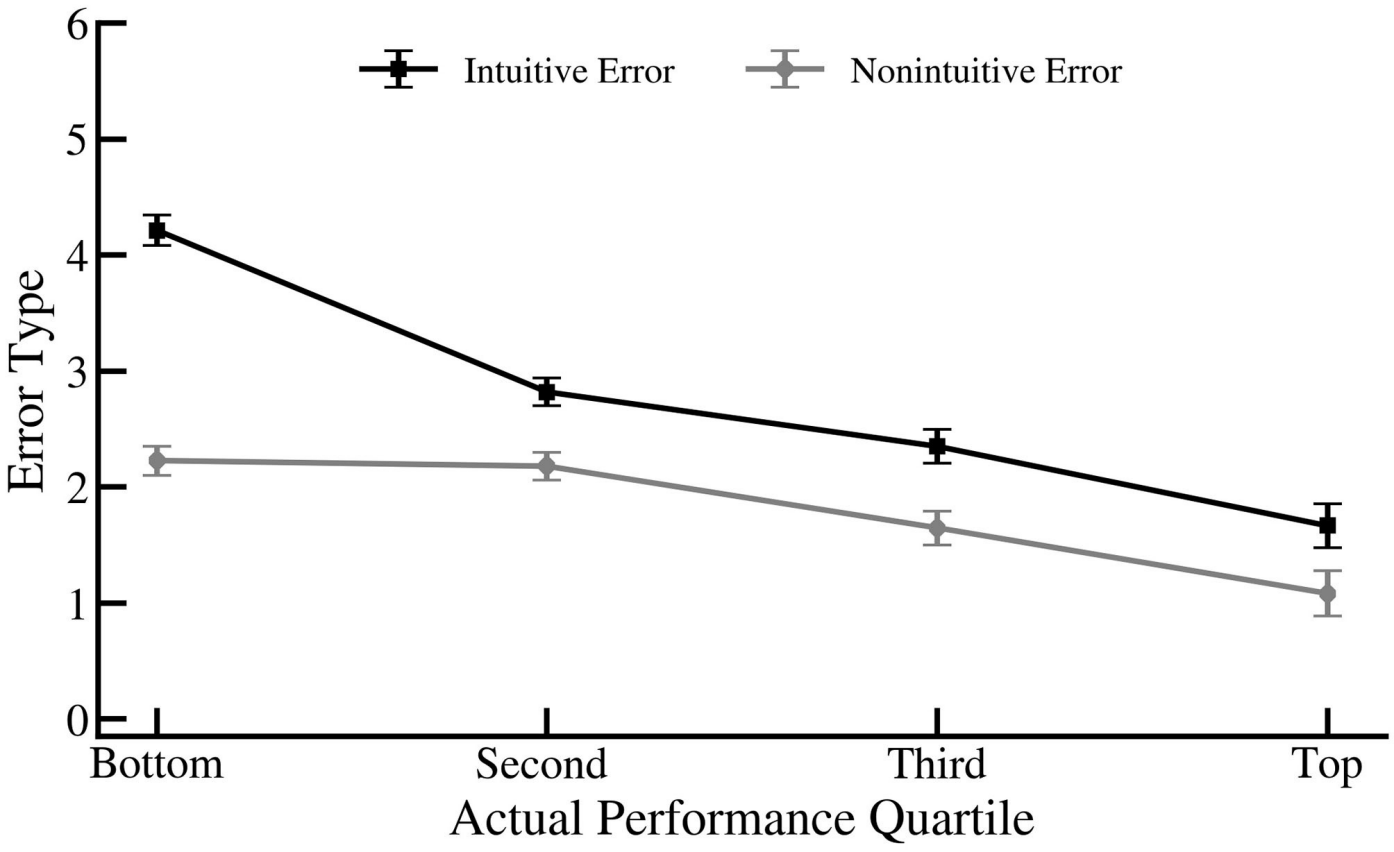
The average number of intuitive and non-intuitive errors for each performance quartile. Error bars indicate standard error.

A quartile ANOVA was performed on error type (intuitive- and non-intuitive-incorrect responses). The ANOVA yielded a main effect of error type, [*F*_(1,165)_ = 30.89], *p* < 0.001, η^2^ = 0.158 and an interaction between quartile and response type, [*F*_(3,165)_ = 7.06], *p* < 0.001, η^2^ = 0.114. Participants in general made significantly more intuitive than non-intuitive errors. But the difference between the number of intuitive and non-intuitive responses was higher for low performers (*M* = 4.21, *SD* = 1.07) than second (*M* = 2.82, *SD* = 0.85, *p* < 0.001), third (*M* = 2.35, *SD* = 0.89, *p* < 0.001), and fourth (*M* = 1.67, *SD* = 0.65, *p* < 0.001) quartiles (See Figure 2).

### Predictors of Miscalibration at Different Performance Levels

To examine the influence of intuitive responses on estimated CRT scores, we simultaneously regressed calibration scores on intuitive- and non-intuitive-incorrect responses for each quartile separately and then for the entire group. With regard to low performers, intuitive responses were positively related to overestimation (b = 0.914, se = 0.273, *β* = 0.819), *t*_(67)_ = 3.35, *p* < 0.001. Non-intuitive errors were also positively related to overestimation (b = 0.660, se = 0.279, *β* = 0.578), *t*_(67)_ = 2.36, *p* = 0.021, *R*^2^ = 0.163, *p* = 0.003, but to a lesser extent than intuitive errors. Thus, while both intuitive and non-intuitive errors predict overestimation among low performers, intuitive errors were shown to be a stronger predictor. Because the top quartile included only 12 participants, we first ran the analysis including data from only the 12 participants and subsequently with data from participants from third and fourth quartiles together (*N* = 49). Neither intuitive- nor non-intuitive-incorrect responses were significant predictors miscalibration among participants in the fourth quartile alone (*p* = 0.120, *p* = 0.232) or third and fourth quartile together (*p* = 0.101, *p* = 0.534), respectively.

In the overall sample, we found that intuitive responding was a better predictor of miscalibration, (b = 0.999, se = 0.072, *β* = 0.805), *t*_(166)_ = 13.83, *p* < 0.001, than non-intuitive responding (b = 0.837, se = 0.094, *β* = 0.515), *t*_(166)_ = 8.86, *p* < 0.001, *R*^2^ = 0.547, *p* < 0.001. Furthermore, we checked the regression models for multicollinearity using variance inflation factor (VIF). The VIF values for the regression models were 4.79 and 1.49 for low performers and for the whole sample, respectively. This shows that multicollinearity was not a problem given that it is <10. These results, taken together, support the hypothesis that intuitive responding is a major factor contributing to miscalibration in the CRT, and that it is particularly a problem for the least capable analytical reasoners.

### Predictors of Estimated CRT Scores at Different Performance Levels

The previous analyses showed that intuitive responding predicts miscalibration among low performers but not high performers. This suggests that bottom performers were less likely to recognize the intuitive responses as incorrect, and hence more likely to factor them into their estimate along with the correct responses, causing them to overestimate. If such a hypothesis is correct, then we would expect that a combined score including intuitive and correct responses would be a stronger predictor of estimated CRT scores than correct responses alone. To test that, we regressed estimated CRT scores on combined intuitive and correct scores for each quartile separately and then for the whole sample. The analysis showed that combined scores were positively associated with estimated CRT scores for low performers only, (b = 0.262, se = 0.127, *β* = 0.244), *t*_(69)_ = 2.07, *p* = 0.042, *R*^2^ = 0.059, *p* = 0.042. All the other analyses were not statistically significant. Furthermore, we looked at whether correct responses alone, or intuitive responses predicted estimated CRT scores. Neither the relationship between correct and estimated CRT Score, *β* = 0.83, *p* = 0.495, nor intuitive and estimated CRT scores were significant, *β* = 0.20, *p* = 0.1. These results show that the best predictor of estimated scores is neither correct responses nor intuitive responses alone, but a combined measure of both responses. This supports the idea that low performers may be especially more likely to perceive the intuitive response as correct.

### Faith in Intuition and Estimated CRT Score

No differences in self-report of intuitive thinking disposition were found across quartiles, despite the fact that low performers had significantly more intuitive responses than any other group. FI was also not related to intuitive responding in the CRT, *p* = 0.284, nor actual CRT scores, *p* = 0.509. However, we found that FI predicted estimated CRT scores, (b = 0.490, se = 0.176, *β* = 0.211), *t*_(167)_ = 2.79, *p* = 0.006. FI also predicted miscalibration when performance was controlled (b = 0.486, se = 0.176, *β* = 0.142), *t*_(167)_ = 2.77, *p* = 0.007, *R*^2^ = 0.20, *p* = 0.007. This indicates that intuitive thinking disposition cannot account for poor performance in general, but it contributes to how participants estimate their performance. That is, people who perceived themselves to be more intuitive tended to give higher estimates of performance and also showed a higher level of miscalibration.

## Discussion

The current study examined the contribution of intuitive errors relative to non-intuitive errors to miscalibration among low and high performers on the CRT. It also tested whether intuitive thinking disposition is related to miscalibration. Consistent with prior studies, we replicated the DKE. Participants with the highest number of errors showed the highest degree of miscalibration. Specifically, on a test that was out of seven points low performers overestimated their CRT score by 4.26, while high performers miscalibrated by just 1. Moreover, low performers made significantly more intuitive than non-intuitive errors, in a ratio much higher than the upper-quartile performers. This suggests that low performers were either (1) less likely to initiate Type 2 processes to verify the correctness of the intuitive response or that (2) initiation of Type 2 processes did not lead to a change of the initial response. This could occur if the output generated by Type 2 processes was not convincing enough for low performers to endorse it.

In support of our hypothesis, intuitive-incorrect responses were stronger predictors of miscalibration than non-intuitive incorrect responses among students in the bottom quartile. Such relationship was not present among high performers. Neither intuitive nor non-intuitive errors predicted miscalibration among those in the top quartile. A possible explanation is that low and high performers do not utilize the same cues, or at least not to the same extent, when judging the accuracy of their responses. Low performers may be more likely to use surface-based cues, such as answer fluency, which are not reliable. This conclusion is consistent with previous research showing differences in cue utilization among students at different levels of performance (Thiede et al., 2010; Ackerman and Leiser, 2014; Gutierrez de Blume et al., 2017). For instance, Ackerman and Leiser (2014) reported that low achievers – when regulating their learning of text—were more sensitive to unreliable, surface-level cues than high achievers. Moreover, Thiede et al. (2010) demonstrated that at-risk readers were more likely to rely on surface-based cues (e.g., ease of processing, readability, length, and specific vocabulary) when judging text comprehension, whereas competent readers based their judgments on valid comprehension-based cues, such as the belief they can explain the text to another person. It is yet unclear why low performers may be more likely to rely on unreliable cues when judging their performance. We argue that limited knowledge or skills, in the domain tested, may be one of the factors driving such bias. For example, limited knowledge or skills could influence cue selection by limiting the number of cues available to the learner, directing the learner's attention to particular unreliable cues, or leading to the formation of erroneous beliefs regarding the validity of cues.

The findings showing that intuitive responses are a stronger predictor of miscalibration than non-intuitive incorrect responses among low performers are in line with our proposal that differences in answer fluency between response types give rise to different metacognitive experiences regarding confidence in the accuracy of the responses, which in turn influence general estimates of performance. As stated earlier, it is possible that low performers interpret increases in fluency as evidence that their intuitive responses are correct, and therefore do not initiate analytical Type 2 processing to double-check. This then causes them to make more intuitive errors and also to count intuitive responses as evidence of good performance when estimating their test score. This argument is in line with previous studies showing that highly fluent responses are endorsed with higher confidence independently of objective accuracy (Thompson et al., 2011, 2013).

Furthermore, when it comes to upper quartile performers, neither intuitive nor non-intuitive errors predicted miscalibration. This finding is probably due to how more analytic- performers interpret answer fluency accompanying intuitive responses. We believe they do not interpret it as an indication that the answer is correct. Thus, when they make an intuitive mistake, they do not endorse it with high confidence. This is in line with previous studies suggesting that analytical individuals have a metacognitive advantage over those who are less analytical given that the former are aware that the intuitive answer is often biased and needs to be verified (Mata et al., 2013).

We also found that self-reported intuitive thinking disposition measured by FI predicted miscalibration. Participants who perceived themselves to be more intuitive were the ones with the largest discrepancy in estimated vs. actual scores. This indicates that people who enjoy and trust their intuition are less likely to recognize when they make a mistake and therefore end up overestimating their performance. This finding is consistent with the main results of the present study showing that responses mediated by Type 1 processes are more susceptible to metacognitive illusions.

### Limitations and Future Directions

The present study included only female participants and 92% were from the UAE. Considering this limitation, we do not know if the outcome of this study would generalize to males in the UAE or to people across cultures. It is however notable that Emirati participants performed similarly to their Western counterparts in other studies. We hope that future research will complement the present study by including participants from both genders and from other cultures. We also did not measure response time for each question in the CRT. A measure of response time would provide us with additional evidence that intuitive responses were indeed produced quicker than analytical correct and non-intuitive incorrect responses. Future studies should consider adding such measure.

It is important to point out that regression to the mean has been shown to explain part of the DKE, but not all (Krueger and Mueller, 2002; Feld et al., 2017). This influence is more prominent for relative judgments than absolute judgments (Dunning, 2011; McIntosh et al., 2019). In our study we used absolute judgments, yet it is possible that regression to the mean had an effect on the degree of miscalibration found and might account for our DKE results. A similar case could be made for motivational biases, wishful thinking, etc. If this were the case, it could have had an indirect effect on the main novel finding that intuitive responses predicted miscalibration in low performers but not high performers. It is unclear whether regression or motivational effects would explain why intuitive responses were stronger predictors of overconfidence than non-intuitive responses. Still, it would have been beneficial if we had attempted to eliminate such artifact by for example using different sets of questions to measure response accuracy and miscalibration (Klayman et al., 1999; Burson et al., 2006; Feld et al., 2017). Similarly, more insight could be gained into the motivational value participants assigned to the task, and whether these can help distinguish between a true DKE and regression to the mean. Additionally, while our Faith in Intuition measure yielded an interesting result that may generalize to other domains, there are a wide variety of other cognitive, social, personal, and motivational factors that might help predict miscalibration that should also be taken into consideration. Finally, more research is needed to explore the link between intuitive responding and estimation error, particularly with regard to the role of answer fluency, and also whether educational training programs could minimize the influence of heuristic-based cues on local and global metacognitive judgments.

The current findings raise an important question: Why do participants with lower scores in the CRT seem more susceptible to low-validity cues, such as answer fluency? One possibility is that when low performers initiate Type 2 processing, the output generated is often wrong due to limitations in the system. Therefore, this causes them to rely more on their intuitions. Because Type 1 processes yield sensible solutions for everyday problems, confidence in them may increase. As confidence in intuitive thinking increases, they rely more on fluency as an indication of successful learning or performance. However, increases in fluency may be caused by factors other than learning and knowing. Relying on fluency thus causes low performers to make several mistakes when judging their performance, and these metacognitive mistakes cause illusory thinking that itself decreases performance and increases confidence. Challenging this faith in intuition could be an important step in correcting the problem. Future research could help low performers to identify and utilize more reliable cues by first demonstrating errors of intuition and then focusing on metacognitive skills. This would be consistent with Schwarz's, Sanna, Skurnik and Yoon (2007) efforts to de-bias people in a variety of domains, in which they note that judgements in one domain (e.g., faith in intuition) can affect judgements in another domain (e.g., performance estimates) even when the two judgements are not directly related. Demonstrating the futility of one domain through educational interventions could help properly calibrate the other.

### Conclusion

We provided evidence that intuitive and non-intuitive errors contribute to miscalibration among low but not high performers on the CRT. Intuitive errors are a stronger predictor of overestimation, and are a particular problem for low performers. This findings provides support to the proposal that low performers' difficulty in recognizing when they make a mistake is partly due to how they interpret variations in fluency accompanying different sources of errors. They seem more likely to view increases in fluency as an indication that an answer is correct, which then lead to inflated self-estimates. Consistent with the main hypothesis, this study also showed that participants who perceive themselves as highly intuitive are more likely to overestimate their performance.

The present study has theoretical and methodological implications to the literature on the DKE, and practical implications to education. First, it suggests that the type of error, whether it is elicited by Type 1 processes, poor numeracy skills or deficient reasoning is likely to give rise to different levels of feeling of rightness among low performers, influencing thus their global self-estimates. Second, it shows that the influence of answer fluency on metacognitive judgments extends to general estimates of performance, and low performers are the ones most susceptible to it. Third, it demonstrates the importance of looking at error type to better understand the mechanisms mediating the DKE. Fourth, it suggests that proper calibration among low performers is unlikely to happen by itself. Hence, education at all developmental levels should directly teach the Dunning-Kruger Effect, problems with intuition, and related cognitive biases. Interventions to improve self-awareness that specifically focus on intuitive biases and fluency may help low performers spot errors, improve metacognitively, and increase performance.

## Data Availability Statement

The dataset for this study can be found in the OSFHOME repository through the following link: https://osf.io/ay23s/?view_only=8803bb83b470401b91dc252b125a86e6.

## Ethics Statement

The studies involving human participants were reviewed and approved by Research Ethics Committee at Zayed University. The patients/participants provided their written informed consent to participate in this study.

## Author Contributions

MC contributed to conceptualization, review of the literature, design and methodology of the study and data collection, data analysis and interpretation, drafting, revision of the draft, and formatting for submission. JT contributed to the literature review, data analysis, and revision of the draft. AA contributed to data collection and the literature review. JC contributed to literature review, drafting, and revision of the draft. All authors contributed to the article and approved the submitted version.

## Conflict of Interest

The authors declare that the research was conducted in the absence of any commercial or financial relationships that could be construed as a potential conflict of interest.
