# Supplementary material for: Dunning-Kruger Effect: Intuitive Errors Predict Overconfidence on the Cognitive Reflection Test
Source: Front Psychol. 2021 Apr 8;12:603225. doi: 10.3389/fpsyg.2021.603225 (PMC8060648; doi:10.3389/fpsyg.2021.603225)
Supplement: Supplementary file 1 [file Table_1.docx]

Supplementary Material

**Table S1**. Means and Standard Deviations for CRT Correct responses, intuitive and non-intuitive errors per Test Item.

| Items | Correct | | Intuitive Erros | | Non-intuitive Errors | |
| --- | --- | --- | --- | --- | --- | --- |
|  | *M* | *SD* | *M* | *SD* | *M* | *SD* |
| 1.If you’re running a race and you pass the person in second place, what place are you in? | .41 | .494 | .54 | .500 | .04 | .200 |
| 2. A farmer had 15 sheep and all but 8 died. How many are left? | .52 | .501 | .41 | .493 | .07 | .258 |
| 3.Emily’s father has three daughters. The first two are named April and May. What is the third daughter’s name? | .50 | .501 | .46 | .500 | .04 | .200 |
| 4.If John can drink one barrel of water in 6 days, and Mary can drink one barrel of water in 12 days, how long would it take them to drink one barrel of water together? | .03 | .170 | .24 | .430 | .73 | .446 |
| 5.Jerry received both the 15th highest and the 15th lowest mark in the class. How many students are in the class? | .05 | .213 | .36 | .480 | .60 | .492 |
| 6. A man buys a pig for $60, sells it for $70, buys it back for $80, and sells it finally for $90. How much has he made? | .14 | .351 | .43 | .822 | .48 | .501 |
| 7. Simon decided to invest $8,000 in the stock market one day early in 2008. Six months after he invested, on July 17, the stocks he had purchased were down 50%. Fortunately for Simon, from July 17 to October 17, the stocks he had purchased went up 75%. At this point, Simon has: a. broken even in the stock market, b. is ahead of where he began, c. has lost money | .13 | .337 | .82 | .383 | .05 | .213 |

**Faith in Intuition Scale Items**

1. I like to rely on my intuitive impressions. (ee)
2. I don't have a very good sense of intuition. (ea-)
3. Using my "gut-feelings" usually works well for me in figuring

out problems in my life. (ea)

1. I believe in trusting my hunches. (ea)
2. Intuition can be a very useful way to solve problems. (ee)
3. I often go by my instincts when deciding on a course of action. (ee)
4. I trust my initial feelings about people. (ea)
5. When it comes to trusting people, I can usually rely on my gut feelings. (ea)
6. If I were to rely on my gut feelings, I would often make mistakes. (ea-)
7. I don't like situations in which I have to rely on intuition. (ee-)
8. I think there are times when one should rely on one's intuition. (ee)
9. I think it is foolish to make important decisions based on feelings. (ee-)
10. I don't think it is a good idea to rely on one's intuition for important decisions. (ee-)
11. I generally don't depend on my feelings to help me make decisions. (ee-)
12. I hardly ever go wrong when I listen to my deepest "gut-feelings" to find an answer. (ea)
13. I would not want to depend on anyone who described himself or herself as intuitive. (ee-)
14. My snap judgments are probably not as good as most people's. (ea-)
15. I tend to use my heart as a guide for my actions. (ee)
16. I can usually feel when a person is right or wrong, even if I can't explain how I know. (ea)
17. I suspect my hunches are inaccurate as often as they are accurate. (ea-)

**____________________________________________________________________________**

Note. Names of the sub-scales that each item belongs are shown in parentheses. ee = Experiential Engagement; ea = Experiential Ability. A minus sign next to the scale name (-) indicates that reverse scoring is required.
